# Friction Behavior between Carbon Fiber Plain Weave and Metal Semi-Cylinder Tool

**DOI:** 10.3390/polym15020472

**Published:** 2023-01-16

**Authors:** Ning Wu, Qi Guo, Ximing Xie, Li Chen

**Affiliations:** 1 School of Textile Science and Engineering, Tiangong University, Tianjin 300387, China; 2 Ministry of Education Key Laboratory of Advanced Textile Composite Materials, Institute of Composite Material, Tiangong University, Tianjin 300387, China

**Keywords:** fabric, pre-forming, friction, contact area

## Abstract

The deformations that occur during composite forming processes are governed by the friction between the fabrics and tooling material on the mesoscopic level. The effect of normal load and multi-plies on the frictional behavior of the carbon plain weave is investigated by simulating the friction between the fabric and metal semi-cylinder tool by using the experimental method. The periodic wavy friction-displacement curve between the metal tool and fabric is caused by the interwoven structure of the fabric. Both the increase in the normal load and the number of layers cause an increase in the real contact area during friction, leading to an increase in the friction force. The real contact area is calculated based on the Hertzian contact model and the self-designed testing method. The friction force values obtained from multiplying the real contact area with shear strength are closely aligned with the measured results.

## 1. Introduction

The growing special demand in many fields such as vehicles, military industry and aerospace, has led to the increasing use of carbon fiber reinforced composite parts due to their high specific strength, highlighted interlaminar shear performance, and high damage tolerance [1,2,3,4,5]. The pre-forming stage is the key link in the manufacturing of carbon fiber reinforced composite parts. According to previous studies [6,7,8], pre-forming progress can cause fabric/fabric and fabric/metal tool friction. It should be emphasized that friction may eventually result in the local defects of carbon fabrics [9,10,11,12,13,14]. Therefore, understanding the friction between the tool and fabric surfaces is critical for the forming quality of the composite parts.

Many different experimental devices and test methods have recently been developed to conduct a series of frictional studies related to fabric. Montero et al. [15,16] employed a plate-friction device to study the friction behavior between fabric layers. The result showed that the typical layer/layer friction behavior for dry carbon fabric is determined by the combined action of tow/tow friction between two fabric plies, and shocks between the interweaving point effect of each ply. In addition, Cornelissen et al. [17] designed a capstan friction device with simple operation. For this method, they measured the friction behavior of carbon fabric and tow on metal and provided a comparison of the frictional behavior of tow and fabric material on different counter faces and loading conditions. Fetfatsidis et al. [18,19] quantified the plain fabric/tool friction behavior using a homemade friction experimental apparatus with adjustable loads. They found that the friction coefficient increased with the increase in velocity and decreased with the increase in normal force and tool temperature.

Knowing the real contact area is essential to understanding and accurately simulating the friction in the forming of fabric composites. Smerdova and Sutcliffe [20,21,22] believed that the real contact area of fabric/tool was composed of fiber/tool, fiber/fiber, and another micro contact. They designed a device for measuring the real fiber/tool contact length. The rig can apply normal loads to the NCF via glass plates and to measure the real contact length of carbon fibers in contact with the plate. Then the contact width between the fiber and the glass plate was estimated by using the Hertzian contact theory, and finally, the actual contact area was obtained. This method was extended by Avgoulas.et al. [23] to study the non-crimp fabric (NCF)/tool contact. It was found that the true fiber contact length of the NCF/tool is reduced by 67% compared to the tow/tool test at 240 kPa. Cornelissen et al. [17,24] used a practical method to calculate the contact area. They put a normal load on the adhesive tape and fabric. The tape was brought into close contact with the fabric, which led to small elliptic areas on the tape. The sum of these elliptic areas was the real contact area.

**Figure 1:**
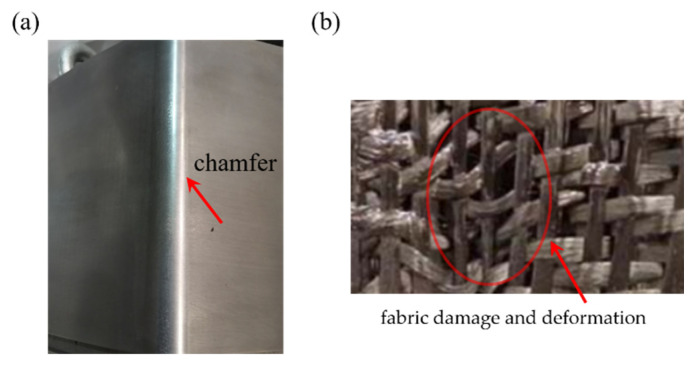
A chamfered mold (**a**) and a deformed fabric (**b**).

In summary, scholars’ studies to date on the friction of carbon fiber fabrics have mainly focused on the frictional behavior between single-layer fabric and fabric or between single-layer fabric and metal under plane contact conditions [15,16,17,18,19,20,21,22,23,24]. In the process of fabric pre-forming, the mold chamfer often comes into contact with the fabric. In the process of fabric pre-form, the contact between the mold chamfer and the fabric will cause some defects of the fabric pre-form, such as fabric wrinkling, yarn slippage, yarn looseness and even breakage. These defects often affect the flow of the resin, which leads to the decline in the mechanical properties of the composites [25,26], as shown in Figure 1. However, the frictional behavior between this metal semi-cylinder surface and multilayer fabrics is far from being entirely understood. The experiment device developed in this study made it possible to simulate the friction behavior of dry fabric composites during pre-forming and to obtain the friction force-displacement curve between the metal tool and multilayer fabrics. In addition, a method was developed to measure the real contact area between the fabric and tool. The Hertzian contact model and Adhesion theory were employed to investigate the relationship between the real contact area and the friction force.

## 2. Experimental Methodology

### 2.1. Friction Test 

**Figure 2:**
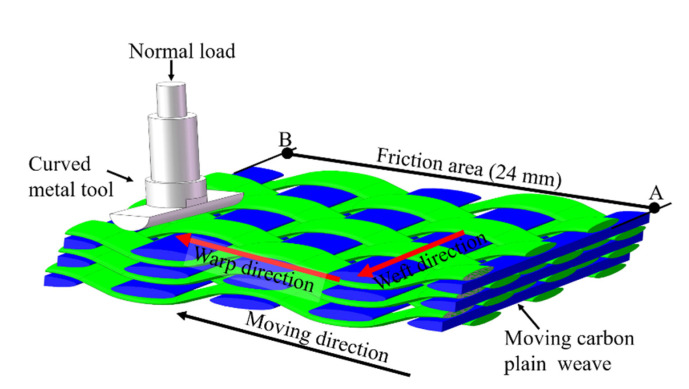
Schematic of one-way motion of the metal semi-cylinder and carbon fabric during the friction test.

Figure 2 is a schematic of the one-way motion of the metal semi-cylinder and carbon fabric during the friction test. During each test, the upper metal semi-cylinder remains stationary, while the lower fabric samples move from point A to point B. The distance between the two points was 24 mm.

**Figure 3:**
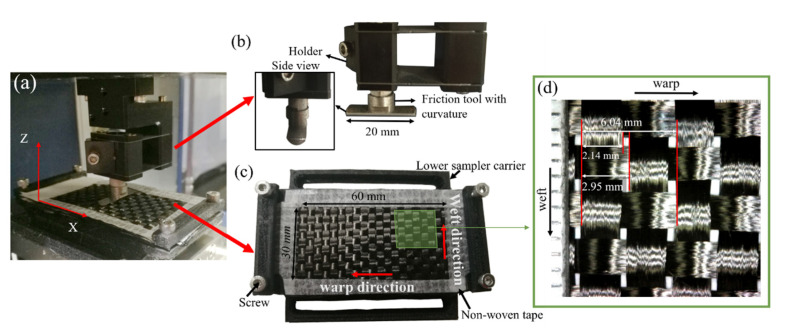
Tribometer of BRUKER (**a**) Photograph of friction test set-up; (**b**) The metal semi-cylinder placed in the upper holder; (**c**) The lower device for fastening fabrics; (**d**) Carbon plain weave for friction test.

The friction measurements between the carbon fiber plain fabric and the metal tool with curvature were performed using a UMT-TriboLab tribometer (Bruker Nano, Inc, Campbell, CA, USA); with this equipment, the set load can be applied accurately within the effective range (0–20 N). It can obtain the force $F_{x}$ in the X axis direction and the force $F_{z}$ in the Z axis direction from the motion process and can calculate the resultant force of the two forces, which is the friction force. Figure 3a shows the photograph of the friction test, which demonstrates that the friction test can be carried out with the help of upper and lower friction devices. The upper friction device in Figure 3b is a semi-cylinder made from ball bearing steels (40 CrMoV), which has a length of 20 mm and a curvature of 0.24. The lower friction device is a self-made fixture, which has the function of fixing the fabric for the friction test. The main body of this device was fabricated by the 3D printer (Raise 3D N2, Inc., Shanghai, China). The four sides of the fabric are affixed by tape to prevent the yarns from detaching from the fabric; then two fastening devices with screws are used to fix the fabric along the warp direction. Figure 3d is a partially enlarged view of the fabric used in the experiment. The carbon plain weave used is made from HF40-12K carbon tows supplied by Hengshen Co., Ltd., Danyang City, Jiangsu Province, China. The weft and warp count is 3.5 yarn/cm and the areal weight is 300 g/m^2^. It is evident from Figure 3d that the width of the yarn is 2.14 mm and the average spacing between neighboring yarns is approximately 2.95 mm. For the fabric samples, the unit cell length is 6.04 mm.

Table 1 illustrates the main parameters of the fabric pre-forms. The friction tests were conducted at five different plies of fabric (1, 3, 5, 7 and 9) that were in the range of the values involved during the dry fabric pre-forming. The normal loads selected were 2, 5, 8, 11 and 14 N which were in the range of the pre-forming for dry fabric composites [27]. The friction speed was maintained at 24 mm/s. All of the tests were carried out in stationary laboratory conditions, the temperature was 27 ± 2 °C and the relative humidity (RH) was 69 ± 3%. The details of the friction experiment parameters are shown in Table 1.

### 2.2. Real Contact Surface Acquisition Method

**Figure 4:**
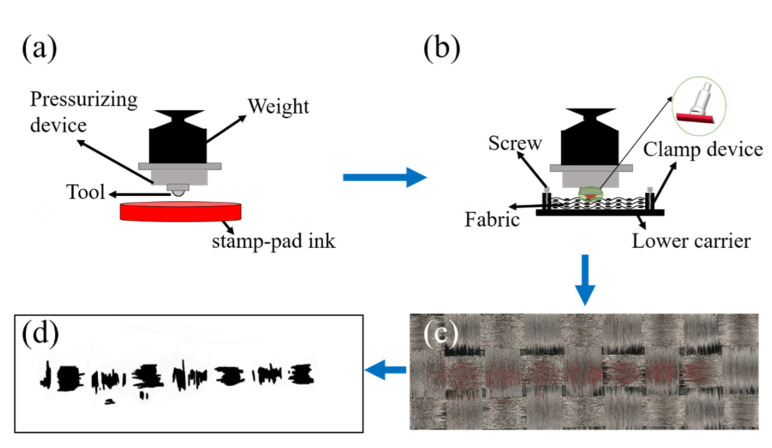
Testing process of contact area: (**a**) Contact between tool and stamp-pad ink; (**b**) Contact between ink and fabric; (**c**) The fabric with stamp-pad ink; (**d**) Binary image of contact area.

The contact area of the friction pairs is considered as one of the most important factors affecting frictional behavior [28,29,30]. However, the warp and weft yarns in the carbon fiber plain fabric are not closely arranged, and the metal tool with a certain curvature cannot complete contact with the carbon fiber plain fabrics. Therefore, in this study, the contact area between the tool and fabric was tested by a self-developed set-up, as reviewed in Figure 4. The contact area test is conducted in four steps: step-1, installing the metal tool on the self-developed pressurization device, and slowly moving the sliding bracket so that the metal tool is in contact with the stamp-pad ink placed directly below, making sure the stamp-pad ink covers the metal tool evenly (Figure 4a); step-2, installing the metal tool with ink on the pressurization device again, moving the sliding bracket to put it in contact with the fabric directly below, adding the weight to make the stamp-pad ink on the metal tool cover the fabric (Figure 4b); step-3, taking out the fabric in the top layers after 15 s (Figure 4c); step-4, transforming the areas of fabric with rubbings into binary images by adjusting the threshold values and then measuring these areas (Figure 4d).

## 3. Results and Discussion

### 3.1. Effect of Normal Load on the Friction Behavior of Fabrics

**Figure 5:**
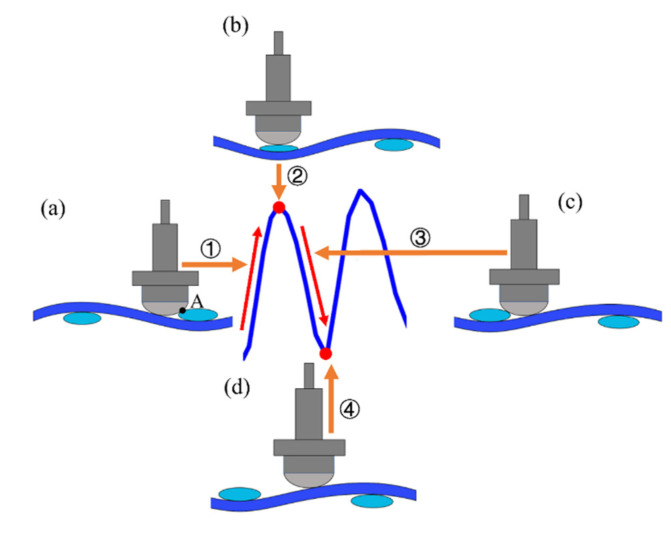
Schematic diagram of the wave-shaped curve generation process: (**a**) Friction increasing process; (**b**) Maximum friction; (**c**) Friction reduction process; (**d**) Minimum friction.

For the curve of the friction force between the metal tool with curvature and the fabric, there will be periodic changes similar to the friction between fabric layers due to the interwoven structure of the fabric. Allaoui et al. [15,16] studied the interlaminar friction of fabrics through self-made experimental devices. The measured friction load-time curve was wavy, and the characteristic period values in the friction test signal were similar to the interval between the adjacent yarns of the fabric. The collision of the interwoven points between two fabric layers leads to the increase in the friction load until it reaches the maximum value, which indicates that the existence of interwoven points will affect the friction behavior between fabrics. Figure 5 illustrates the generation process of the wave-shaped curve. As shown in Figure 5a, the raised interweaving points hinder the advancement of the metal tool, causing the friction force to soar. When the metal semi-cylinder moves to the top of the interweaving points, the maximum friction force is formed (Figure 5b). Consequently, the friction force gradually decreases as the contact position is away from the interweaving point (Figure 5c). When the tool moves into the middle of the neighboring weft and warp interweaving points (Figure 5d), the impact of the shock completely disappears; then, the friction force drops to the minimum.

**Figure 6:**
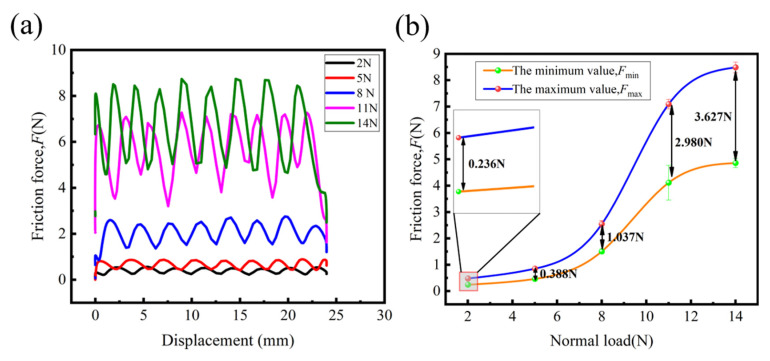
Effect of normal load on the friction force of fabrics: (**a**) Friction-displacement curves; (**b**) maximum value ($F_{max}$) and minimum value ($F_{min}$) with increasing loading force.

Figure 6a shows the effect of normal load on the friction force of the fabrics. The friction force was performed using a UMT-TriboLab tribometer, as shown in Figure 3. As seen in Figure 6a, the curves of the friction force fluctuate in a manner akin to waves, and the overall level of the friction curve increases with the increasing normal load. The distance between the adjacent maximum and minimum values of each cycle is approximately 3 mm, which is similar to the distance between the adjacent warp yarns (or weft yarns) in Figure 3d, which proves that the interwoven fabric structure gives the friction force-displacement curve wavelike cycle characteristics. Moreover, the corresponding curve waveforms in Figure 6a change from uniform (2–8 N) to unstable (11 N) to uniform (14 N). This is most likely because when the normal load is 8 N, it is close to the critical value of compression deformation at the interweaving point. However, when the normal load is further increased to 11 N, the loading force breaks through the critical value, which leads to the deformation of the surface interweaving point structure, and the friction force-displacement curve presents an unstable waveform. When the normal load increases to 14 N again, the deformation degree of the surface interweaving point structure reaches the maximum, and the friction force-displacement curve returns to the uniform waveform.

Figure 6b shows the average maximum(minimum) values of the tool/fabric friction force under different normal loads, and the difference between the maximum and minimum values is marked. It can be seen that with the increase in the loading force, the difference between the maximum value and the minimum value increases. There is a rapid growth trend between 8 N and 11 N, which demonstrates, from the side, that the continuous increase in the normal load will lead to the deformation of the fabric interweaving points. When the normal load increases to 14 N, the deformation degree reaches the maximum, while the maximum and minimum values of the friction force gradually stabilize and the difference between them also remains stable. In addition, compared with the other loads, the standard deviation of the minimum value of the friction force at 11 N is larger, which indicates that the dispersion degree of the minimum value at 11 N is higher. This corresponds to the instability of the friction force-displacement curve at 11N in Figure 6a, and further indicates that the trend of the friction force-displacement curve under different normal loads shifts from uniform (2–8 N) to unstable (11 N) to uniform (14 N).

### 3.2. Correlation Analysis of Number of Plies, Real Contact Area and Friction Force 

**Figure 7:**
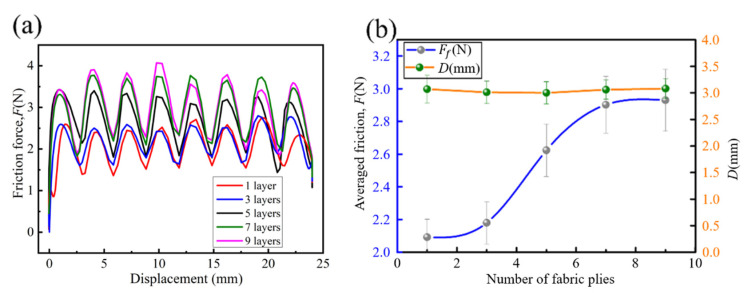
Effect of number of plies on friction force: (**a**) Friction force-displacement curve; (**b**) Curves of averaged friction and fabric plies.

The effect of the number of plies on the friction force is shown in Figure 7. As can be seen from Figure 7a, the friction curves maintain a waveform with almost equal spacing between the crests and valleys. This law repeats as the friction displacement increases. It follows from Figure 7b that the frictional force increases with the number of layers of the fabric, particularly between three and seven plies, which produces a rapid increase. The growth of the friction force increases by 20.34% with the increasing number of layers from three to five, while by merely 0.0345% with the increasing number of layers from seven to nine. The distance (D) between the neighboring maximum and minimum values of each cycle remains constant (~3 mm). This means that D is insensitive to the number of plies.

**Figure 8:**
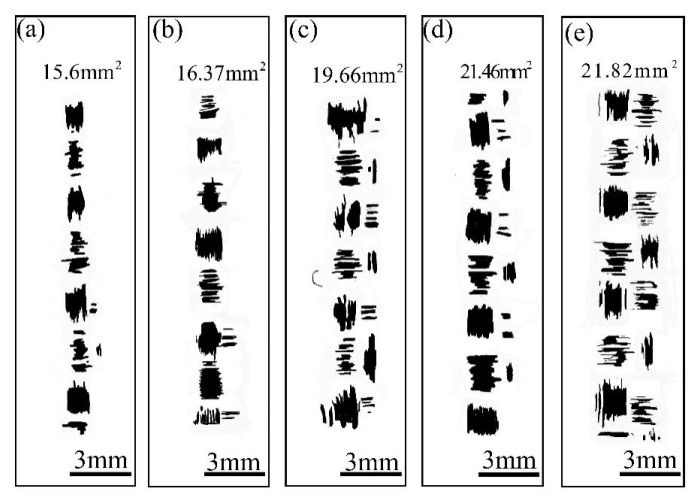
The morphology of contact area between metal tool and fabric with different plies: (**a**) 1 ply; (**b**) 3 plies; (**c**) 5 plies; (**d**) 7 plies; (**e**) 9 plies.

The contact area between the metal tool and the fabrics with different layers can be obtained by using the measurement method of the real contact area in Section 2.2. The morphology of the contact area between the metal tool and the fabric in the range of 1, 3, 5, 7, 9 plies (8 N, 24 mm/s) is illustrated in Figure 8. From Figure 8 it can be observed that the area of the colored part of the fabric increases with the increasing number of layers. This is because the fabric tends to deform, with the same force producing a greater deformation of the multi-layer fabric surface, increasing in the real contact area. This also is also the reason the spacing between the frictional wave maximums and minimums becomes wider with the increase in layers, as it aggravates the impact between the metal tool and the interweaving point.

**Figure 9:**
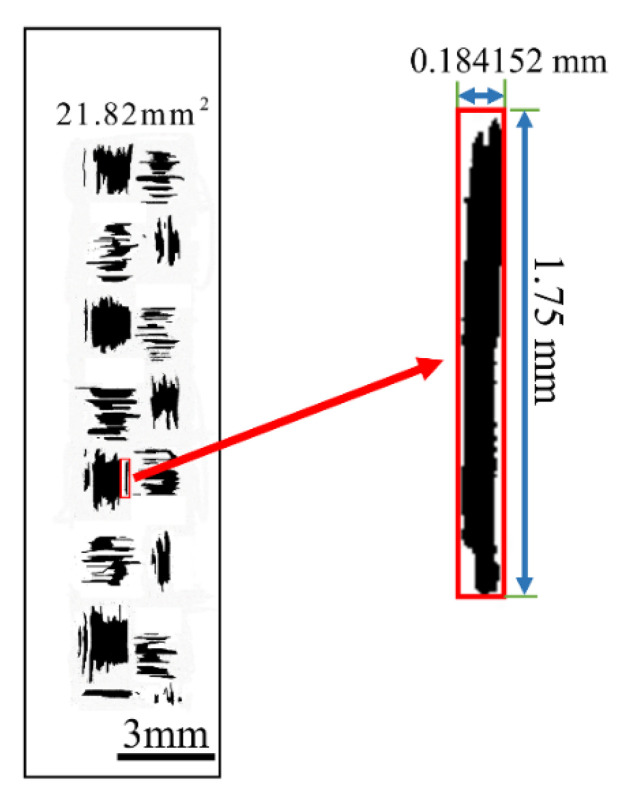
Real contact area statistics method.

Undoubtedly, the biggest milestone in contact mechanics is Hertz’s research on static elastic contact. The Hertzian contact theory is based on the elastic theory, and it is still the basis of the elastic contact force models available in the literature. In addition, the Hertzian contact theory can easily incorporate friction models [31]. In essence, the contact behavior between the tool/yarn is equivalent to that between the tool/filament. Thus, this contact behavior can be simplified to cylinder/plane, by the Hertzian contact theory in three steps: step-1, figuring out the real contact area of red rectangle (Figure 9); step-2, figuring out the ratio of the real contact area in the red rectangle to the red rectangle area (λ); step-3, obtaining the real contact area with different plies by λ (%).

Based on the Hertzian contact theory, the real contact area between the metal tool and the fabric can be given by the following equations:

(1) $$a={\left(\frac{2Nd}{\pi E^{*}L}\right)}^{\frac{1}{2}}$$

(2) T=1.494g

(3) $$E^{*}=\frac{1-V_{tool}{}^{2}}{E_{tool}}+\frac{1-V_{carbon}{}^{2}}{E_{carbon}}$$

(4) $$A_{0}=2aT$$

(5) $$\lambda =\frac{A_{0}}{A_{j}}$$

(6) $$A_{r}=\lambda A_{m}$$

where a is the contact half-width (mm), N is the normal load (800 mN), d is the diameter of carbon filament (4.5 μm), T is the total length of carbon filaments in the rectangular area (μm), g is the number of carbon filaments in the rectangular area (24 counts), $E^{*}$ is the material equivalent elastic modulus (134.62 Gpa), $E_{tool}$ is the elastic modulus of metal semi-cylinder (210 Gpa), $E_{corbon}$ is the elastic modulus of carbon fiber (294 Gpa), $A_{o}$ is the real area in the red rectangle (mm^2^), $A_{j}$ is the area of the red rectangle (mm^2^), $A_{r}$ is the real contact area of tool/fabric (mm^2^), $A_{m}$ is the mesoscopic contact area of tool/fabric (mm^2^).

**Figure 10:**
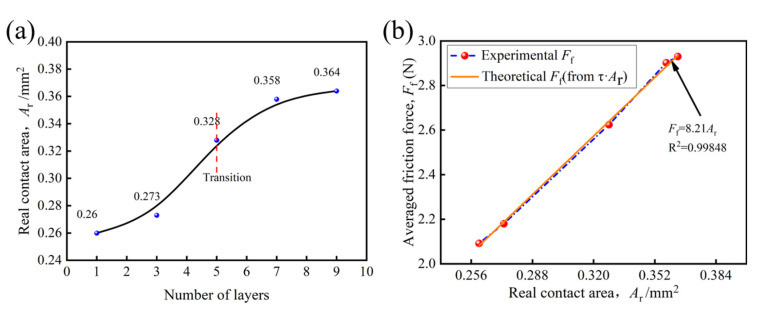
Real contact area: (**a**) real contact area of tool/fabric with different plies; (**b**) Measured friction force $F_{f}$ versus real contact area $A_{r}$. Real contact area $A_{r}$ times a suitable shear strength τ yields a curve which closely overlays the measured friction force F.

The results of the contact area statistics are plotted in Figure 10a. As shown in Figure 10a, the $A_{r}$ increases as the number of ply increases. The variation of $A_{r}$ ranges between 0.26 and 0.364 mm^2^. An interesting phenomenon is that, as the number of plies increases, the real contact area shows a similar trend to the friction force in Figure 7b, both showing a rapid increase when laying 3–7 plies, the real contact area hardly changes between seven plies to nine plies. 

Figure 10b demonstrates both the measured friction force curves and the curves obtained from multiplying the real contact area $A_{r}$ by a suitable constant τ. $F_{f}$ increases with a growth of $A_{r}$, and this relationship is approximately linear. In other words, the frictional behavior of the tool/fabric holds for the adhesion theory ($F_{f}=\tau A_{r}$). Thus, the linear fitting equation can be given as:

(7) $$F_{f}=8.21A_{r}$$

According to Equation (7), the shear strength (τ) carbon plain weave in this paper is 8.21 MPa. Due to the frictional sub-conditions and other factors, there is still no uniform standard for the selection of the τ values for carbon fiber materials [22,24]. Adhesion theory holds that an increase in $A_{r}$ enhances the adhesion of the tool/fabric, which leads the friction force to increase [20,32]. It should be stressed that factor $A_{r}$ increases with the increasing plies, as is confirmed by Figure 10a. In conclusion, the increase in plies leads to the increase in the real contact area, which intensifies the adhesion of the friction pair and finally leads to the growth in the averaged friction force ($F_{f}$).

## 4. Conclusions

The aim of this investigation was to understand the fabric friction phenomenon in the pre-forming stage of dry fabric composites. An experimental simulation device was developed to reproduce the friction behavior between the multi-layer carbon plain weave and the metal mold with curvature.

The effect of a normal load on fabric/tool friction have been investigated. The results show that the overall level of the friction curve increases with the increasing normal load. The typical friction force curve characters generated by the friction of the metal semi-cylinder and carbon plain weave has been investigated. The wavelike cycle characteristic curve of the friction force-displacement is caused by the interwoven structure of the fabric. Furthermore, the greater the normal load, the more significant the difference between the maximum and minimum friction force is.

The relationship between the number of plies, the real contact area, and the friction force has been established. The increase in the plies leads to the increase in the real contact area between tools and fabrics. The results of the fit show a linear relationship between the growth of $F_{f}$ and $A_{r}$, on the fabric surface, which is in accordance with the adhesion theory model.

## Figures and Tables

**Table 1:** Friction experimental process feature parameters.

Sample Number	Loading Force (N)	Number of Layers
F1	2	5
F2	5	5
F3	8	5
F4	11	5
F5	14	5
F6	8	1
F7	8	3
F8	8	5
F9	8	7
F10	8	9

## Data Availability

The data presented in this study are available on request from the corresponding author.
